# Coronary Computer Tomography Angiography in 2021—Acquisition Protocols, Tips and Tricks and Heading beyond the Possible

**DOI:** 10.3390/diagnostics11061072

**Published:** 2021-06-10

**Authors:** Sorin Giusca, Moritz Schütz, Florian Kronbach, David Wolf, Peter Nunninger, Grigorios Korosoglou

**Affiliations:** 1 Department of Cardiology, Angiology and Pneumology, GRN Hospital Weinheim, 69469 Weinheim, Germany; sorin.giusca@grn.de (S.G.); moritz.schuetz@grn.de (M.S.); Florian.Kronbach@grn.de (F.K.); david.wolf@grn.de (D.W.); 2 Cardiac Imaging Center Weinheim, Hector Foundation, 69469 Weinheim, Germany; 3 Practice of Radiology, GRN Hospital Weinheim, 69469 Weinheim, Germany; peter@nunninger.de

**Keywords:** CCTA, prognosis, chronic coronary syndromes, review, plaque analysis, diagnosis coronary artery disease

## Abstract

Recent technological advances, together with an increasing body of evidence from randomized trials, have placed coronary computer tomography angiography (CCTA) in the center of the diagnostic workup of patients with coronary artery disease. The method was proven reliable in the diagnosis of relevant coronary artery stenosis. Furthermore, it can identify different stages of the atherosclerotic process, including early atherosclerotic changes of the coronary vessel wall, a quality not met by other non-invasive tests. In addition, newer computational software can measure the hemodynamic relevance (fractional flow reserve) of a certain stenosis. In addition, if required, information related to cardiac and valvular function can be provided with specific protocols. Importantly, recent trials have highlighted the prognostic relevance of CCTA in patients with coronary artery disease, which helped establishing CCTA as the first-line method for the diagnostic work-up of such patients in current guidelines. All this can be gathered in one relatively fast examination with minimal discomfort for the patient and, with newer machines, with very low radiation exposure. Herein, we provide an overview of the current technical aspects, indications, pitfalls, and new horizons with CCTA, providing examples from our own clinical practice.

## 1. Introduction

Coronary artery disease (CAD) remains an important cause of morbidity and mortality worldwide [1]. Currently, the term “chronic coronary syndromes” (CCS) gathers under one umbrella all patients who are affected from CAD in different forms and stages, excluding those with acute coronary syndromes (ACS) [2]. The work-up of patients with CCS is complex, beginning with the evaluation of the pre-test probability for CAD and proceeding with the selection of appropriate treatment pathways. In this regard, non-invasive stress imaging tests were shown to be excellent tools for properly selecting patients who would further benefit from an invasive procedure, thus providing both precise diagnostic classification and risk stratification [3,4]. 

Among all non-invasive diagnostic methods employed in the work-up of patients with CCS, coronary computer tomography angiography (CCTA) stands out as an excellent integrative tool that provides information regarding calcium burden, presence and degree of coronary stenosis, type of atherosclerotic plaques, and functional relevance of such plaques, all in one examination [5,6]. In addition, newer scanners that can acquire high-contrast images with very low doses of radiation (<1 mSv) allow for the performance of longitudinal studies and thus provide valuable data regarding the response of the atherosclerotic plaque to specific therapies [7]. 

Moreover, in the era of the ORBITA and ISCHEMIA trials, the optimal therapeutic approach for patients with significant CAD became a subject of debate, and in many situations shared decision approaches are recommended [8,9]. In this regard CCTA presents itself not only as an excellent diagnostic tool but also as an educational tool for the patient. The relatively “simple” representation of the coronary arteries provides the patient with a clear view and with the help of the physician, an understanding of the underlying anatomical problems.

## 2. Population Selection

### Who Should Get a CCTA Scan?

The technological advances seen in the last decades, together with a growing body of research confirming the relevance of this diagnostic method in the clinical routine, have made CCTA a first-line diagnostic tool for the diagnostic work-up of CAD patients [10]. These developments were paralleled by an increasing acceptance and consequently higher levels of recommendation from societal guidelines, changes that were seen in the last years as new data from randomized trials became available. In this regard, the NICE (National Institute for Health and Care Excellence) guidelines were the first societal guidelines who positioned CCTA as a first-line and gatekeeper examination for patients with stable symptoms and a suspicion of CAD [10]. This was followed by the recommendations of European Society of Cardiology for the diagnosis and treatment of patients with CCS, which also placed CCTA as a first line diagnostic tool with a level of recommendation I [2]. It thus become appropriate to perform a CCTA for most of the patients encompassing the entire spectrum of CCS, including even those patients with known CAD, previous coronary artery bypass operations (CABG), or stent implantation [11]. Furthermore, CCTA is a valuable diagnostic method for evaluating patients prior to non-cardiac surgery [11]. In addition, to increase the specificity of the method, fractional flow reserve derived with CCTA (FFR_CT_) can be employed for the evaluation of the functional significance of coronary artery stenoses [12]. 

The diagnostic performance of CCTA was also confirmed in patients with acute symptoms and suspected acute coronary syndromes (ACS) [13]. Patients who present with acute chest pain in the emergency department can have a plethora of underlying causes. Together with a thorough clinical examination, electrocardiogram (ECG), and laboratory values, CCTA can help in reaching an expedite diagnosis and thus provide appropriate care for the patients [14,15,16]. Thus, CCTA is now recommended in patients with ACS at low to intermediate likelihood of CAD without a relevant increase in troponin levels as a first diagnostic line and was recently shown to reduce the number of unnecessary invasive procedures in the VERDICT trial [17,18]. Moreover, special protocols such as triple rule-out (TRO) can confirm/infirm other life-threatening conditions apart from CAD, such pulmonary embolism or aortic dissection [19]. 

The evaluation of asymptomatic patients with CCTA still remains an unelucidated question. Although the assessment of calcium scoring (CAC) was shown to significantly improve the risk stratification of such patients and a CCTA might be useful for patients with relevant cardiovascular risk factors, no prognostic data are available in this population and thus no clear recommendation can be made [20,21]. Possibly, the undergoing SCOT-HEART 2 Study (ClinicalTrials.gov Identifier: NCT03920176) will provide answers to this question.

## 3. Technical Aspects

### 3.1. What Do We Need?

Moving coronary arteries are challenging structures to visualize non-invasively. This is related to their relatively small diameter and their continuous movement throughout the cardiac cycle. The ideal diagnostic tool for optimally depicting the coronary arteries should be able to synchronize to the cardiac cycle (“gating”), visualize structures at sub-millimeter resolution (“spatial resolution”), and acquire the entire dataset at time frames of a couple dozens of milliseconds (“temporal resolution”) [22]. With the introduction of the 64-slice CT scanners, these requirements were met at such degree that the visualization of the coronary arteries was feasible with diagnostic image quality [23]. Since then, several technical advancements in hardware and software further improved the diagnostic accuracy of CCTA. In this regard, the introduction of dual-source CT translated in an improvement in temporal resolution from 125–175 to 65–75 ms. Furthermore, developments in detector technology allowed for the covering of the entire length of the heart in a single heartbeat [24]. Even though most modern scanners exhibit excellent image quality with high isotropic spatial (best 0.35 mm) and temporal (best 65 ms) resolutions, these parameters are still half of what is needed and compared to the resolution provide by invasive coronary angiography (ICA), (0.2 mm and 30 ms, respectively) [22,24]. Developments in hardware technology were mirrored by advances in image processing software. Thus, as the computation power increased in the last two decades, images are currently generated using “iterative reconstruction” in comparison to the conventional “filtered back projection”. This allows for a reduction of noise in the image and also reduces the need for strong currents when the acquisition is performed, therefore minimizing the radiation exposure for the patients [25,26]. 

### 3.2. Which Protocol Needs to Be Chosen?

**Figure 1:**
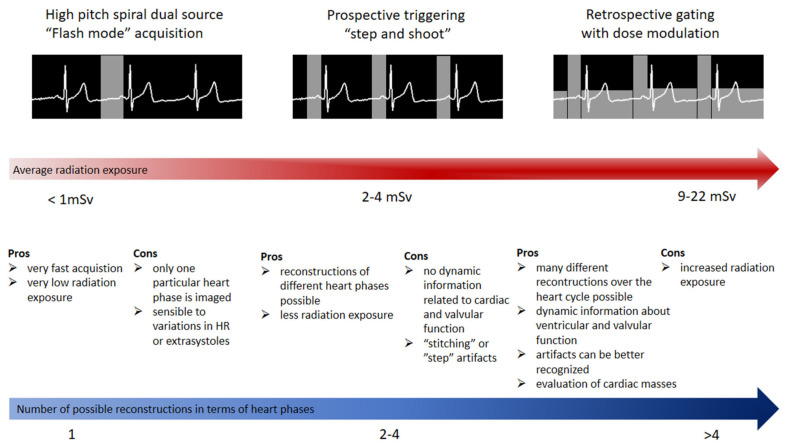
Scan modes used in coronary computer tomography angiography with advantages and disadvantages as well as radiation exposure for each of the different acquisition approaches.

**Figure 2:**
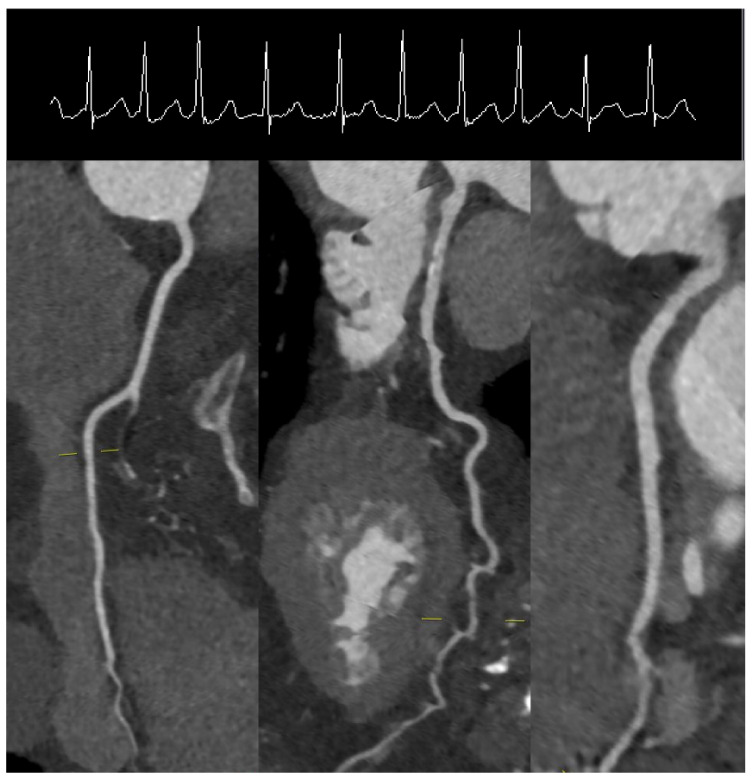
“Step and Shoot” protocol triggering during systole in a patient with atrial fibrillation and a heart rate of 114/min.

**Figure 3:**
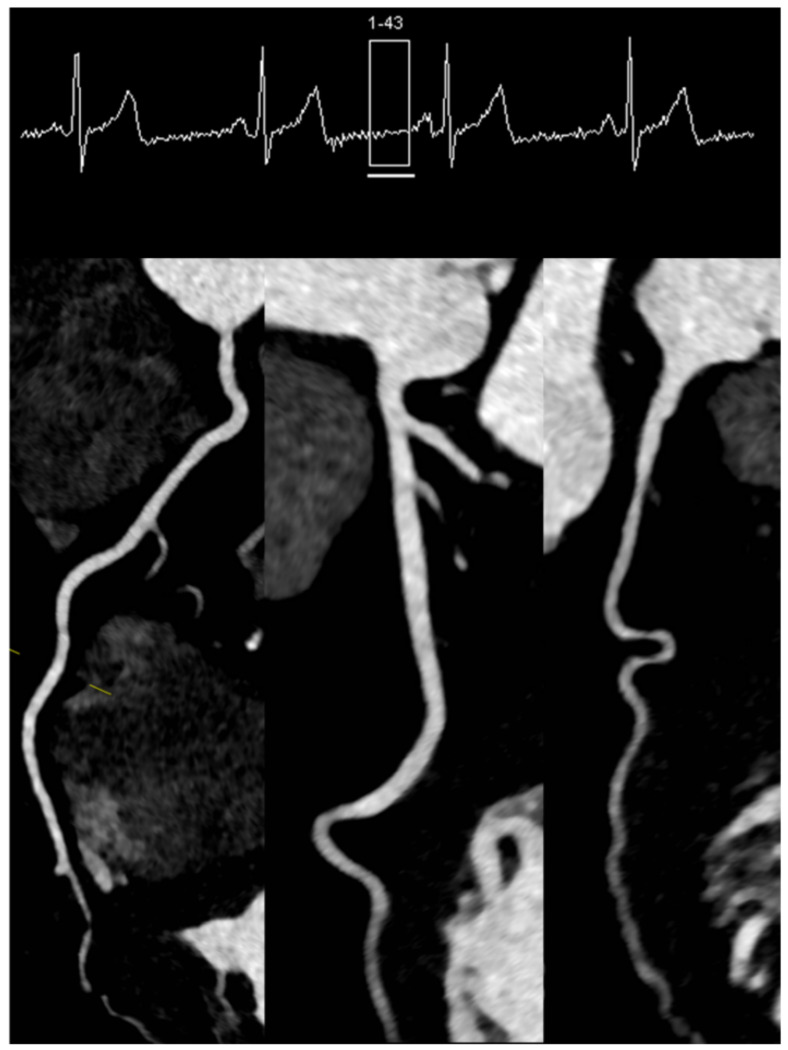
High-pitch dual source spiral protocol (“Flash” mode) in a patient with a stable heart rate of 58/min.

Modern scanners are versatile in regard to the type of protocol employed for acquiring the data [27]. This should always be tailored to the specific individual (i.e., habitus, heart rate, irregularities in heart rate, etc.) and the specific clinical question (status of the coronary arteries, left ventricular function, follow-up CCTA, etc.). Generally, three types of ECG-gated protocols can be employed during a CCTA acquisition [28] (Figure 1). The retrospective ECG-gated acquisition in “helical” or “spiral” mode was the first type of acquisition used in CCTA [29]. When retrospective gating is used, data are acquired during the entire cardiac cycle. The main advantage of this protocol is the ability to reconstruct CCTA acquisitions at various time points of the cardiac cycle. Furthermore, it provides information related to cardiac volumes and function as well as valvular anatomy and function. The main disadvantage is related to the high radiation exposure for the patient. Thus, currently, even when this type of protocol is chosen, a dose-modulation algorithm is used, which reduces the tube current to around 20% outside the time frames chosen for imaging the coronary arteries. Even at this low radiation dose, the evaluation of cardiac and valvular function is still possible. The analysis of the motion of the coronary arteries during the cardiac cycle revealed that these vessels exhibit the lowest movement and thus optimal time frame for acquisition during end-systole (30–40% from the RR cycle) or/and mid-diastole (60–70% from the RR cycle) [30]. Thus, it is conceivable that the image acquisition should optimally occur within these time windows. Keeping that in mind, the next protocol that was developed was the prospectively ECG-triggered axial acquisition [31]. With this scan mode, also known as “sequential” or “step and shoot”, images can be obtained without table movement during acquisition. However, as most of the detectors are smaller than the length of the heart, several heartbeats are needed for a complete coverage of the heart. This type of acquisition usually provides images with excellent contrast and with relatively low radiation exposure [32,33]. The drawbacks of such a protocol, however, are mainly represented by a relatively reduced number of possible reconstructions of cardiac phases as well as the presence of “stitching” or “step” artefacts, especially when heart rate variability is present between the different stacks. A very useful application of this protocol is in patients with irregular heartbeats and/or atrial fibrillation. In these patients, the diastole varies significantly from beat to beat, which would make the acquisition of the data in diastole relatively useless [34]. However, as the coronary arteries show reduced movement at end-systole as well, and the systole is less affected by irregularities in heart rate, a “step and shoot” acquisition triggered during the systole often provides excellent image quality (Figure 2). Lastly, the technical advances seen in the last decade have enabled the development of protocols that allow the sampling of data needed for a full characterization of the coronary tree during a single heartbeat. This can be achieved in two ways, depending on the scanner. In scanners that allow a detector coverage of >=16 cm, a complete dataset can be acquired without moving the patient and during a single heartbeat, depending on the heart rate [35,36]. Conversely, with dual-source scanners, an ECG-triggered high pitch “helical” or “spiral” acquisition (a pitch value of ≈3) or so-called “Flash” mode allows for the entire dataset to be sampled in one heartbeat [37,38]. This type of acquisitions offers very high contrast images without “stitching” artefacts with very low radiation exposure for the patient (usually <1 mSv) [39] (Figure 3). The main drawbacks of this acquisition mode, however, are related to high dependency on heart rhythm and the limited ability for image reconstruction only during a single time point. 

### 3.3. How Much Can We Reduce Radiation and Contrast Agent Exposure?

Radiation and contrast agent exposure constitute the main risks of a CCTA. With regard to radiation exposure, the radiation received by the patient is mainly dependent on the protocol employed and patient’s habitus. A retrospective ECG-gated protocol using a single source 64-slice CT yields radiation doses between 9 and 22 mSv [40,41]. A significant reduction in radiation exposure can be achieved with prospectively ECG-triggered protocols. Thus, in such patients, the mean effective dose can be as low as 1.2 mSv [40,42]. A meta-analysis including over 3000 patients comparing the radiation exposure between retrospective helical and prospective ECG-triggered protocols confirmed a fourfold dose reduction (from 12.3 to 3.5 mSv) with prospective acquisitions [43]. Lastly, the radiation exposure for the patient can be further minimized using prospective ECG-gated high-pitch acquisitions. In most of these patients, a mean effective dose of <1 mSv can be reached [39]. It is very important to tailor a specific protocol to the clinical question. Although high-pitch acquisitions are very promising in regard to image quality and radiation exposure, the examination is still highly dependable on the heart rate and stability of the heart rhythm during the acquisition. Thus, this type of protocol is usually well suited only in selected patients, especially in those who undergo follow-up examinations during pharmacologic treatment. Several strategies can be employed for reducing the radiation exposure to the patient [44]. For patients, in whom a retrospective protocol is employed, using ECG-dependent tube current modulation can have a significant impact on the amount of radiation received, while the ability to judge valvular and myocardial function is simultaneously maintained [45,46]. However, the coronary arteries can be usually analyzed only in the reconstruction obtained from the timeframes where the maximum current is applied. A further strategy involves reducing the tube voltage (from 120 kV to 100 kV or 80 kV). This has the advantage of reduced effective dose for the patient while increasing the opacification of the coronary arteries [31,47]. However, this strategy may not be so suitable for patients with a BMI > 30 Kg/m^2^ due to the radiation scatter effect [44]. 

The application of contrast agent can have adverse effects on the renal and thyroid function, and the function of these two organs should be evaluated prior to performing the CCTA. Patients with diabetes and a reduced renal function defined as eGFR < 45 mL/min/1.73 m^2^ are at higher risk for developing contrast-induced nephropathy [48]. Newer scanners can acquire high quality datasets with relatively low amounts of iodine contrast (50–60 mL) and in selected populations can be as low as 30 mL by acquiring at low tube voltages [49,50]. Two methods can be employed to determine the optimal time point for the acquisition: bolus tracking and test bolus. Although each method has advantages and disadvantages, the test bolus method can better help identifying more accurately the optimal moment for acquisition [51,52]. An important aspect of contrast agent administration is to ensure an opacification of coronary vessels between 250 and 350 Hounsfield units (HU), since values outside this range may prevent the accurate evaluation of coronary stenoses [53]. 

## 4. Diagnosis

### 4.1. Do We Still Need Calcium Scoring?

**Figure 4:**
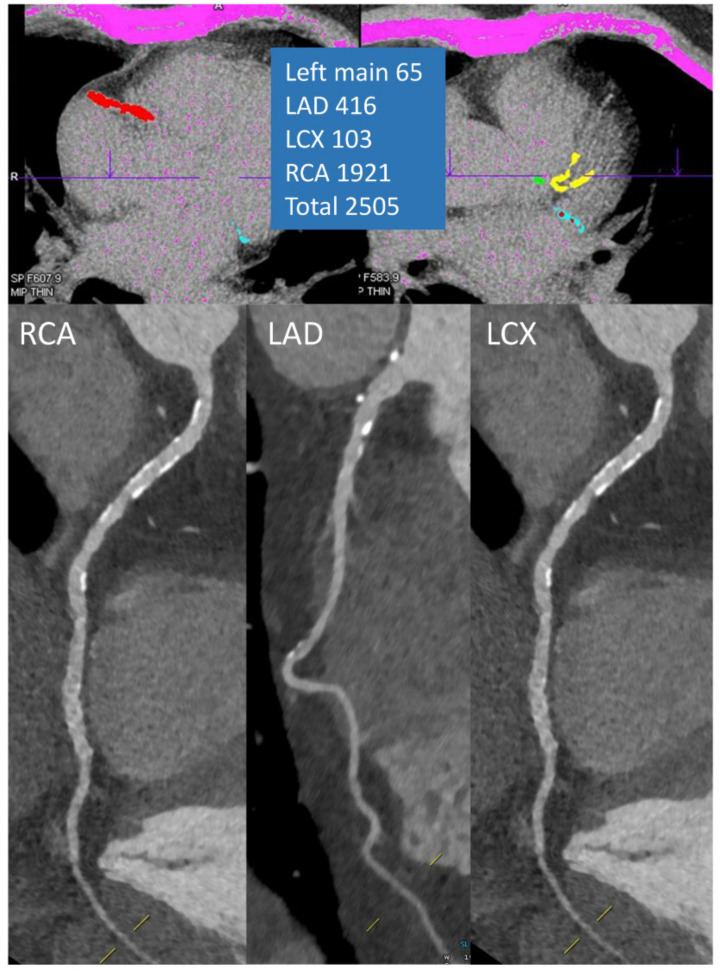
Excellent image quality in a patient with a very high CAC score.

A hallmark of atherosclerosis is the presence of calcium deposits. Thus, the identification of calcium in the coronary arteries correlates with the atherosclerotic burden of coronary arteries [54,55,56]. The specific acquisition for determining the amount of calcium in the coronary arteries usually takes place in the first phase of the CCTA and consists of non-contrast, non-overlapping axial slices with an individual slice thickness of 3 mm, acquired in the mid-diastole using ECG gating triggering [57]. CAC is defined on the reconstructed images as an area of hyper-attenuation of at least 1 mm^2^ with an intensity >130 HU in three adjacent pixels [58]. The most used method for quantification is the Agatston-system, which grades the presence of calcium by multiplying the area of calcification with a factor corresponding to maximum plaque attenuation as follows: 130–199, factor 1; 200–299, factor 2; 300–399, factor 3; above 400, factor 4 [58,59]. The prognostic value of CAC in the asymptomatic population has been demonstrated in numerous studies encompassing over 50,000 individuals [60,61]. CAC has consistently exhibited significant predictive power for future cardiovascular events, with incremental value to conventional cardiovascular risk factors, including the Framingham risk score. Thus, a CAC of 0 is associated with a low event rate even in the presence of conventional cardiovascular risk factors—event rate of 2.72 per 1000 person-year in patients with three or more risk factors [62]. Conversely, in patients with a CAC > 400 and no risk factors, the annual event-rate per 1000 asymptomatic individuals was 16.89 [62]. The main clinical value of CAC lies in the ability to reclassify asymptomatic individuals with an intermediate risk for cardiovascular events on the basis of conventional risk assessment [63,64]. In this regard, almost two thirds of patients in an intermediate bracket based on the Framingham scale would be reclassified when taking into account the CAC. This reclassification value is maintained also in the high-risk group, where a third of patients would be reclassified by CAC [63]. Current European guidelines on cardiovascular disease prevention recommend the use of CAC screening with a IIb indication as a possible risk modifier in cardiovascular risk assessment [65]. Care must be taken, however, when interpreting the CAC, since low-attenuating plaques will not be identified, and a high calcium burden does not automatically translate into the presence obstructive CAD (Figure 4). Still, it maintains a significant value in starting or adjusting preventive strategies, such as optimization of the lipid profile and lifestyle interventions as well as in the appropriate planning of the CCTA acquisition. 

### 4.2. Stenosis Visualization and Quantification: The “Heart” of the Problem

**Figure 5:**
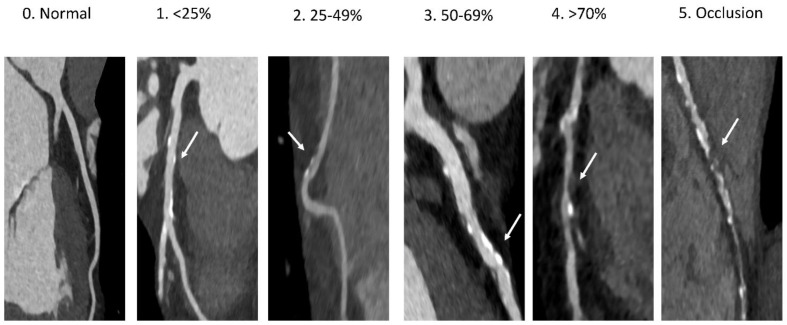
Examples of stenosis grading from 0–25% to 100% occlusions.

CCTA has been extensively compared to invasive coronary angiography for the diagnosis of luminal coronary stenosis [66,67]. The results of the studies are consistent in pointing to an excellent sensitivity and negative predictive value [68,69,70]. This translates in a reduced need for invasive procedures in patients with CCS. However, the positive predictive value in identifying a 70% stenosis can be as low as 48% [69]. Of note, most of the studies were performed in the era of the 64-slice multidetector CT scanners. Newer technologies such as duals source and 320 detector-row led to significant improvements of the positive predictive value of an anatomically significant stenosis up to 80% [71,72]. In regard to stenosis grading, an important aspect of CCTA has to be taken into account when reporting the severity of the stenosis, namely, that the spatial resolution of the most scanners is still half of that provided by invasive coronary angiography (see above). When that is kept in mind, an exact quantification of luminal stenosis generally is not recommended. Thus, reporting of individual stenosis should be based on the “range” of the stenosis as follows: normal—absence of plaque and no luminal stenosis, 1: minimal—plaque with <25% stenosis, 2: mild—plaque with 25–49% stenosis, 3: moderate—50–69% stenosis, 4: severe—70–99% stenosis, 5: occluded (Figure 5) [73]. In addition, the Coronary Artery Disease Reporting and Data System (CAD-RADS™) provides a by patient classification of CAD, considering the most severe stenosis present. This classification encompasses information related to plaque characteristic as well as the presence of stents and bypass grafts (Table 1) [74]. The latest expert consensus of the Society of Cardiovascular Computer Tomography recommends this classification for use in clinical routine [11]. 

In addition, if modifiers are present, they should be added at the end of the classification. G is graft, S is stents, and V is plaque vulnerability.

#### 4.2.1. Pitfalls in the Evaluation of Coronary Stenoses: What Should We Look Out for?

**Figure 6:**
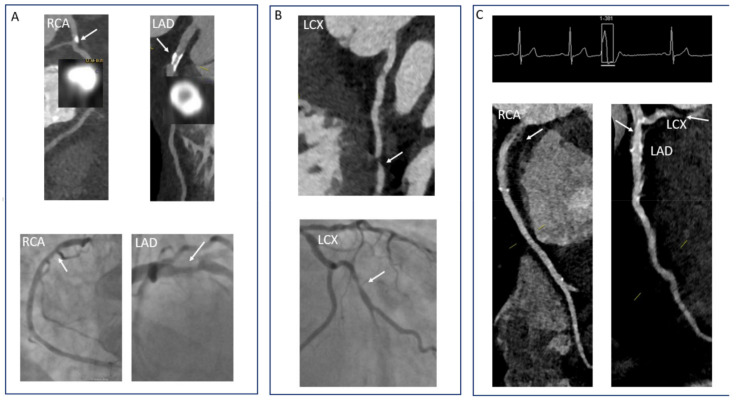
Pitfalls in the interpretation of CCTA images. (**A**) Severe localized calcification can hamper an accurate assessment of the level of stenosis. In this case, CCTA overestimated the stenosis in the RCA and underestimated the stenosis in the LAD (images from ICA for comparison). (**B**) Step or stitching artefacts can impede the accurate assessment of the vessel, especially if a stenosis is located at the level of the step. In this case, a moderate stenosis of the LCX was difficult to evaluate (ICA images for comparison). (**C**) An extrasystole at the moment of the acquisition can make segments of the coronary arteries uninterpretable when using a high-pitch protocol.

The relatively low positive predictive value can be traced to several pitfalls in interpreting CCTA acquisitions [75]. Severe calcifications represent a main obstacle in interpreting the degree of stenosis in a coronary vessel. This is mainly related to the blooming artefact, which leads to an inaccurate estimation of the disease severity (Figure 6A). Thus, the accuracy of CCTA significantly drops if the analyzed segments show a calcification greater than 50% of lumen diameter [76]. Several strategies can be employed to circumvent this limitation. Firstly, a sharper kernel can be used for the reconstruction of the dataset and thus help reduce the blooming artefact [77]. Furthermore, a stenosis is very likely <50% if contrast is present adjacent to an eccentric calcified plaque [78]. A higher BMI (>30 Kg/m^2^) could reduce the accuracy of a CCTA scan, mainly due to reduced signal to noise ratio secondary to increased X-ray scatter. Several approaches such as increased tube voltage, very good heart rate preparation, and administration of contrast with a higher rate (up to 7 mL/s) can improve the quality of the CCTA [79]. Of note, the ACCURACY study did not identify high BMI as a predictor of reduced sensitivity and specificity [69]. Depending on the protocol used, step or stitch artefacts can also reduce diagnostic image quality [75]. This becomes mainly problematic, when a stenosis is located exactly at the level of the step (Figure 6B). Selecting the appropriate protocol depending on the heart rate variability is therefore of paramount importance to avoid these types of problems. When the heart rate is very stable and <65/min, a high-pitch spiral protocol might be more appropriate. When many extrasystoles are present or the heart rate shows high variability such as in atrial fibrillation, acquisitions in systole should be favored. Of note, the high-pitch spiral protocol is not without drawbacks, even when the patient has a low heart rate with a minimal heart rate variability. Thus, the occurrence of an extrasystole at the moment of the acquisition usually makes the dataset uninterpretable (Figure 6C), and thus a repeated scan using a different protocol is necessary in such cases. Lastly, care should be taken when analyzing certain segments of the coronary arteries, which have a very curved trajectory. This applies for the distal segment of the RCA and origin of the posterior descending artery, the proximal segment of the left anterior descendent artery with the origin of the first diagonal branch, and origin of the first obtuse marginal branch [77].

#### 4.2.2. CCTA versus Other Diagnostic Modalities: Which One to Choose?

The diagnostic value of CCTA was compared to other non-invasive functional tests in several studies. Thus, the EVINCI study evaluated 475 patients with stable chest pain with CCTA, stress perfusion imaging, and wall motion imaging by either stress echocardiography or stress cardiovascular magnetic resonance imaging and used the results of invasive coronary angiography as reference standard. CCTA provided the best accuracy in identifying hemodynamically relevant CAD (area under the curve (AUC) 0.91) compared to all other methods [80]. These data are further supported by a meta-analysis comparing CCTA to SPECT, which showed higher sensitivities (99% vs. 71%) and specificities (71 vs. 48%) for CCTA in identifying relevant CAD [81]. A more recent study compared CCTA and SPECT for the diagnosis of CAD in 391 patients. They found similar results with CCTA exhibiting higher accuracy for the diagnosis of relevant CAD in comparison to SPECT (AUC of 0.92 vs. 0.64 for stenosis detection >50%) [82]. In addition, costs represent a major factor when adopting new diagnostic and therapeutic approaches. Ideally, a new medical tool should not result in increased costs. The last major trials involving CCTA evaluated the financial aspect of performing a CCTA. In the PROMISE trial, there was no significant difference in costs in the short term (<90 days) and long term (3 years) between the CCTA arm and the functional test arm [83]. The SCOT-HEART trail identified slightly increased costs for the CCTA arm at 6 months [84]. However, the same trial demonstrated a significant reduction in hard endpoints at 5 years of follow-up in the CCTA arm, which may largely compensate the initially increased costs in the long-term. The CRESCENT trial, on the other hand, found lower costs in the CCTA arm in comparison to the functional testing arm, mainly due to a reduction in terms of downstream testing [85]. 

### 4.3. Plaque Characteristics: Are All Plaques Created Equally?

**Figure 7:**
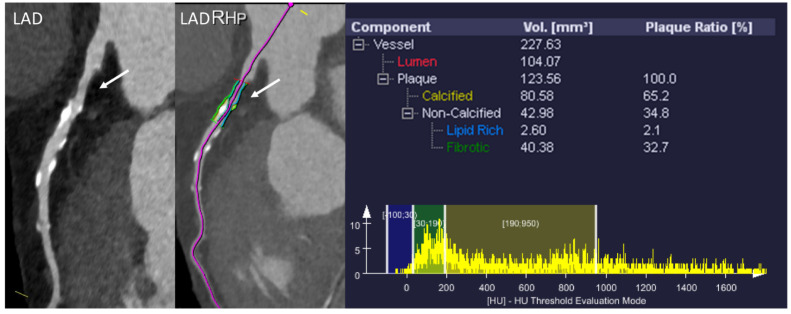
Example of plaque analysis (research software: syngo.via Frontier Coronary Plaque Analysis, Siemens Healthineers). Analysis performed in a moderate plaque in the proximal segment of the LAD. Note the presence of a partially calcified plaque with a predominance of calcium and a low percentage of lipids.

A major advantage of CCTA over conventional ICA is its ability to non-invasively provide detailed characterization atherosclerotic plaque composition and volume (Figure 7). This is a unique feature for a non-invasive test, and an entire body of research and literature has focused on this issue in the last decade [6]. A quick overview of the atherosclerotic process points the role of wall shear stress (WSS) at the beginning of such pathophysiologic processes, explaining why many of the coronary plaques are seen in regions with an increased shear stress such as bifurcations. This is followed by the accumulation of low-density lipoprotein molecules in the endothelial cells, which activate an inflammatory process [86]. The inflammatory process is further augmented and so-called “vulnerable” plaques develop, which are characterized by necrotic cores and thin fibrous cap [87]. However, not all plaques take this pathway, and “stable” plaques, which have a low percentage of necrotic core and high percentage of calcium deposits, are also frequently seen. The exact mechanisms that underpin the development of a specific type of atherosclerotic plaque are not yet completely understood. 

**Figure 8:**
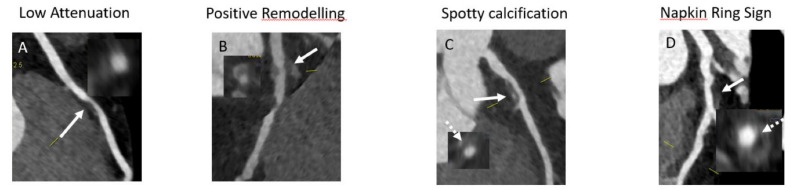
Examples of high-risk plaques. (**A**) Low-attenuation plaque (white arrow). (**B**) Positive remodeling—note the development of the plaque outside the coronary lumen (white arrow). (**C**) Spotty calcification—note the presence of calcium in an otherwise low-attenuation plaque (white arrow). (**D**) Napkin-ring sign—a heterogenous low-attenuation plaque (white arrow) that exhibits a cap with lower HU densities (interrupted white arrow) in comparison to the rest of the plaque.

CCTA is an excellent tool for identifying various stages of the atherosclerotic process and especially for identifying vulnerable plaques, which are considered as precursors of ACS. On the basis of the amount of calcium present, we can classify plaques as calcified, partially calcified, and non-calcified [88]. Several studies have looked at the correlation between calcium composition and cardiovascular endpoints. Although the correlation with the cardiovascular endpoint was significant in univariate analysis in most studies, with non-calcified plaques exhibiting precursors of ACS and cardiac mortality, when models were adjusted for conventional risk factors, the significance of the correlation sometimes became less relevant [89,90]. Thus, it appears that classifying plaques only based on the amount of calcium present might be too “simplistic” and not provide enough prognostic information [91,92]. In this regard, a closer look at plaque morphology and composition and comparison with histopathological data revealed several patterns consisting of high-risk features of coronary atherosclerotic plaques in CCTA [93,94]: low attenuation, positive remodeling, spotty calcification, and napkin-ring sign (Figure 8):

- Low attenuation plaques are considered to mirror the vulnerable plaques characterized by necrotic lipid rich core. As CCTA can distinguish between lipid and fibrotic tissue in terms of HU, it is conceivable that analyzing plaques in terms of HU can identify those with a predominant lipid composition. In this regard, several studies used intravascular ultrasound (IVUS) as reference standard for the characterization of low attenuation plaques [95,96]. Although significant overlap was seen, a value of <30 HU provided identification of vulnerable lipid-rich plaques with good sensitivity and specificity [97,98].
- Positive remodeling is a process that occurs in the early stages of atherosclerosis and is considered as a compensatory mechanism of the vessel to maintain a sufficient non-stenotic area in the context of atherosclerotic plaque progression [99]. The presence of positive remodeling is associated with a lipid-rich plaque and accumulation of macrophages and necrotic tissue [100]. A 10% increase in the diameter at the level of the stenosis in comparison to the reference diameter outside the stenosis is considered to indicate positive remodeling [101]. Similar to all other features of high-risk plaque, positive remodeling is seen more often in patients with ACS compared to patients with CCS [102].
- Spotty calcifications are more commonly seen in plaques with thin fibrous cap than in stable plaques [103]. Microcalcifications are considered to be a promoter of plaque destabilization and were often identified in culprit lesions of patients with ACS [104].
- The napkin-ring sign is a form of low attenuation plaque that exhibits a heterogenous pattern of attenuation. Thus, the core shows low attenuation pattern indicative of lipid rich necrosis and the cap displays high attenuation indicative of the fibrotic cap [105]. This type of pattern is highly suggestive of vulnerable plaque and is considered as a precursor of plaque rupture [106]. The high predictive value of the napkin-ring sign for future ACS is quite consistent across studies [107,108,109].

The role of high-risk features in predicting future cardiovascular events was confirmed in several trials. The PROMISE trial highlighted the relevance of high-risk plaque features for predicting cardiac events [110]. In the same line, high-risk plaque features were predictive of the endpoint in the SCOT-HEART study [111]. Furthermore, high-risk plaque features were shown to correlate with troponin releases in patients with stable angina, thus suggesting that silent plaque ruptures with micro-embolization can occur in patients deemed as “stable” [112]. In addition, troponin and high-risk plaque features offer additive value in predicting future cardiovascular events [113]. 

### 4.4. Plaque Stabilization and Regression: Can We Turn Back the Time?

The role of lipid-lowering therapies in the primary and secondary prevention of patients with CAD is widely recognized [114,115]. HMG-CoA reductase and newer molecules such as PCSK9 inhibitors or small interfering ribonucleic acid have all exhibited significant effects on reducing cholesterol values and improving the prognosis of patients with CAD [115]. The first data in regard to the effect on plaque burden were provided by IVUS. Thus, patients who received high-dose statin therapy exhibited a significant reduction in plaque burden in the ASTEROID and SATURN trials [116,117]. The REVERSAL trial, on the other hand, found a reduced progression in atheroma burden in patients receiving atorvastatin compared to those receiving pravastatin [118]. These results were confirmed by a meta-analysis that included over 7000 patients [119]. However, IVUS still remains an invasive approach burdened by possible complications. In this regard, CCTA represents an excellent monitoring tool for the serial non-invasive assessment of atherosclerotic burden. Newer machines that provide very good image quality at sub-millisievert radiation exposure appear in particular to be very appropriate for this purpose. Most of the data currently available in regard to the evolution of plaques analyzed with CCTA stems from small studies with various methodologies [120]. The results are conflicting, and this may be related to the type of study, used methodology, and low number of patients. Nevertheless, a more recent study, involving 467 patients who received LDL-lowering therapies and who were followed up at 2 years with CCTA, highlighted a significant plaque burden reduction in patients who reached a target LDL < 70 mg/dl [121].

### 4.5. Perivascular Fat: Identifying the Problems before They Even Start?

Besides lipids, inflammation plays a crucial role in the development of the atherosclerotic lesion, especially in the early stages. Peri-coronary adipose tissue (PCAT) appears to play an active role in the atherosclerotic process [122]. Thus, Goeller et al. found that PCAT attenuation is increased around culprit versus non-culprit lesions in patients with ACS [123]. When CCTA is used, a fat attenuation index (FAI) can be derived that expresses weighted attenuation shifts within the perivascular tissue [124]. When measured in the proximal segments of the coronary arteries (i.e., the first 40 mm of the RCA, LAD, and LCX), FAI was shown to mirror the inflammatory burden of the entire coronary tree, detecting early subclinical CAD [125]. Furthermore, in the CRISP-CT study including over 4000 patients the FAI measured around the proximal segment of the RCA exhibited a strong predictive power in regard to all-cause and cardiovascular mortality, improving the predictive model containing conventional cardiovascular risk factors, plaque burden, and high-risk plaque features [126]. 

### 4.6. Computational Fluid Dynamics and Fractional Flow Reserve CT: Moving from Anatomy to Hemodynamic Significance

**Figure 9:**
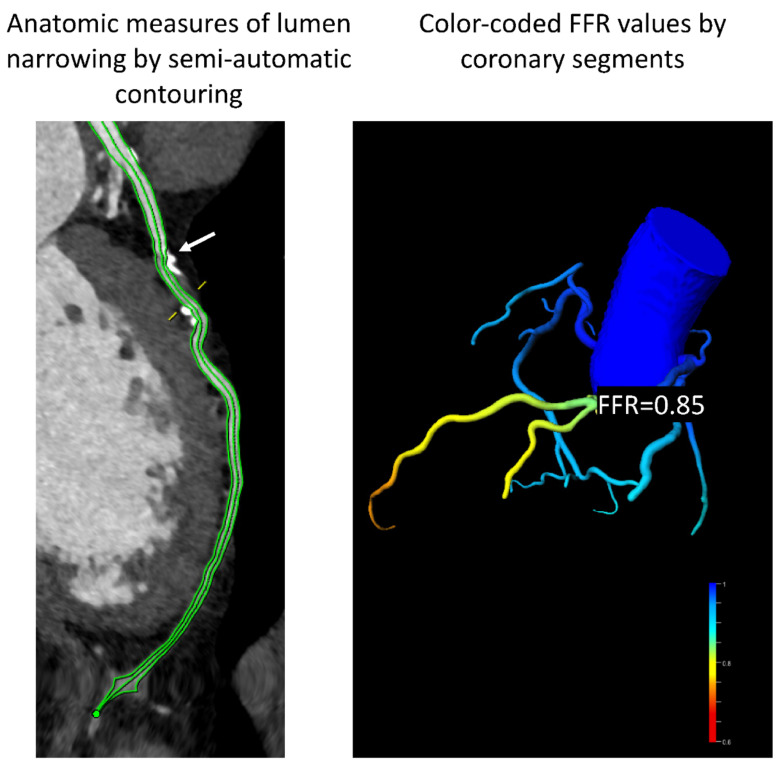
Example of FFR_CT_ analysis (research software: syngo.via Frontier cFFR, Siemens Healthineers). The analysis is used for scientific and not for clinical purposes. Analysis performed on the entire coronary tree. Note a moderate plaque in the proximal LAD, which revealed a FFR_CT_ value of 0.85.

The FAME studies elegantly showed that patients undergoing PCI exhibit a benefit in terms of cardiovascular endpoints only when hemodynamically relevant stenoses (FFR < 0.8) are treated [127,128]. Thus, testing the functional significance of a stenosis plays a major role in establishing the appropriate treatment in these patients. The reference standard for measurement is the invasively obtained parameter under vasodilator stress derived from intracoronary pressure wire measures [129,130]. Until a decade ago, CCTA could only offer very detailed anatomical information. Recently, however, using computational flow dynamic algorithms applied to standard CCTA datasets, FFR_CT_ values across the entire coronary tree can be derived using a commercially available software [131]. The main advantage of this method is its ability to estimate hemodynamic significance of coronary lesions without the need for additional acquisitions during pharmacologic stress (Figure 9). The ability of FFR_CT_ to accurately identify hemodynamically relevant stenoses was tested in several studies that used invasive FFR as the reference standard. In this regard, the DISCOVER-FLOW study was the first study to find a good accuracy (AUC of 0.9) between FFR_CT_ and invasive FFR [132]. These results could be confirmed in the NXT trial, which also found a good accuracy (AUC 0.9) and increase in specificity (79% vs. 34%) of FFR_CT_ compared to standard CCTA for the identification of hemodynamically relevant lesions in 254 patients [133]. Furthermore, the PLATFORM study demonstrated a reduction in the need for coronary angiography in the arm where the FFR_CT_ measurements were used for clinical decisions, without a change in terms of prognosis between the two study arms [134]. In addition, a sub-study of the PACIFIC trial evaluated FFR_CT_ against invasively measured FFR as well as PET and SPECT and found superior AUC in the diagnosis of relevant coronary stenoses compared to standard CCTA, PET, and SPECT [135]. All these studies were performed with the currently only commercially available software and authorized for clinical diagnosis HeartFlow FFR_CT_ (HeartFlow, Redwood, CA, USA). As clinical experience increases with this method, several approaches related to interpreting data from FFR_CT_ should be taken into consideration. Thus, it appears that due to model constraints, very distal segments could exhibit values <0.8 without the presence of a hemodynamically relevant stenosis. In addition, values between 0.75 and 0.8 lie in a gray area and should be clarified using additional testing [136]. 

Other computational methods were developed to extract hemodynamic data from three-dimensional CCTA datasets. The virtual functional assessment index (vFAI) can be employed in establishing the functional relevance of a coronary stenosis. It was firstly developed from ICA datasets and essentially mimics the invasively obtained FFR values [137]. The vFAI can also be obtained from CCTA datasets and was shown to exhibit very strong correlation with invasively measured FFR [138]. Furthermore, when compared to PET, the addition of vFAI to conventional CCTA increased the diagnostic accuracy for identifying impaired vasodilation capacity [139]. An integrative approach in modelling risk stratification, diagnosis, treatment, and prognosis of patients with CAD is represented by the SMARTool [140]. Clinical data, anatomical information, and data extracted using various computational models such as vFAI are fed into a decision support system. When this method is used, CAD could be predicted with an accuracy of 83% [141]. 

Another function of computational fluid dynamics is to clarify the underlying mechanical mechanisms that determine the evolution of coronary plaques [142,143]. Thus, it was shown that coronary segments with low wall shear stress (WSS) exhibited a more accelerated plaque progression in comparison to those segments with intermediate and high WSS [144,145]. Furthermore, in comparison to patients with intermediate WSS, patients with low and high WSS had a higher progression of the necrotic core [145]. Similar results were obtained in the PREDICTION study that showed that segments with low WSS progress towards higher plaque burden and lumen narrowing [146]. Most of these data are, however, obtained using ICA. The integration of CCTA data in the analysis of fluid dynamics has revealed a significant association between higher plaque burden and regions of the coronary tree with low WSS [147]. On the other hand, regions with high WSS appear to be associated with plaques exhibiting high risk features as well as with culprit lesions in patients who develop acute coronary syndromes [148]. 

### 4.7. Perfusion Imaging in CCTA: Not Just the Coronaries

CCTA offers another approach in establishing the relevance of a coronary stenosis besides FFR_CT_, namely, the opportunity to perform myocardial perfusion under hyperemia (CTP). The principle is similar to that employed in other non-invasive stress tests such as CMR, SPECT, or PET and is based on inducing “forced” vasodilation of the distal arterioles using hyperemia inducing agents such as adenosine, dipyridamole, or regadenoson [149,150]. Several protocols have been proposed such as dynamic versus static scanning or stress-first versus rest-first scanning, each one exhibiting specific advantages and disadvantages [150]. Dynamic CTP might offer a more quantitative and thus objective approach in establishing myocardial perfusion, but this comes at a higher radiation cost for the patient since serial acquisitions are needed. The stress-first approach was proven to increase the sensitivity of the method; however, a rest-first acquisition can exclude from the beginning a relevant CAD and thus make a stress CTP unnecessary [151]. The value of CTP in the work-up of patients with known or suspected CAD was tested in several studies. Thus, the CORE320 study tested the diagnostic value of combining CCTA and CTP in identifying stenosis of the coronary arteries >50% and using the results of the SPECT examination as the standard of reference. A total of 381 patients were evaluated, and the study found an AUC for CCTA-CTP in identifying relevant CAD of 0.93 [152]. Furthermore, the addition of CTP increased the diagnostic accuracy of CCTA. The PERFECTION study compared the diagnostic accuracy of CTP vs. FFR_CT_ on top of standard CCTA using invasively obtained FFR as standard of reference in 147 patients. The authors found similar diagnostic accuracies of CCTA-CTP vs. CCTA-FFR_CT_ for identifying relevant CAD. Furthermore, both CTP and FFRCT improved the diagnostic strength of CCTA alone [153]. Moreover, the use of CTP was shown to improve the diagnostic accuracy of CCTA alone in 150 patients with previous stents by identifying relevant in-stent restenosis as shown by the ADVATNAGE trial [154]. 

## 5. Prognosis: Can We See into the Future?

There is mounting evidence related to the prognostic value of CCTA. Perhaps the most representative analysis in this regard was the SCOT-HEART trial [155]. In this study, 4146 patients were randomized to a standard of care arm (2073 patients) or to a standard of care including the performance of a CCTA (2073 patients). At five years of follow-up, the CCTA arm exhibited a reduced event rate regarding cardiovascular death and myocardial infarction in comparison to the standard arm of care (48 patients vs. 81 patients, *p* = 0.004). Although patients in the CCTA arm had more invasive procedures in the first two years, at the five-year follow-up no difference in the number of invasive procedures was noted between the two arms. The SCOT-HEART study was a landmark study for CCTA, and with regards to this study, CCTA was found to be the only non-invasive diagnostic method with the “back-up” of a randomized trial that had a positive effect on prognosis when used in the routine work-up of patients with known or suspected CAD. Another important study was the PROMISE trial [156]. In this study, over 10,000 patients were randomized to either a functional testing arm (exercise electrocardiography, nuclear stress testing, or exercise echocardiography) or anatomical testing arm (CCTA). At a two-year follow-up, the study did not identify a significant difference in terms of prognosis between the two arms. However, data from the PROMISE trial revealed an important difference between functional testing and CCTA in regard to prognosis [157]. Thus, patients with a normal or mildly abnormal functional testing result still have an event rate of 2.09%, and the discriminatory power of functional testing in terms of prognosis begins only in patients with moderate ischemia. CCTA on the other hand, has an excellent discriminatory power at every stage of disease, with a normal CCTA translating into an exceptionally low event rate of 0.93 and a mildly abnormal CCTA an event rate of 3.01 (c index 0.72 for CCTA vs. 0.64 for functional testing). Other studies, albeit non-randomized, also highlighted the prognostic value of CCTA [158,159]. Furthermore, it appears that the prognostic relevance of CCTA is maintained over a long period of time [160]. 

## 6. Special Scenarios

### 6.1. Evaluation of Stents: Can We See through Metal?

**Figure 10:**
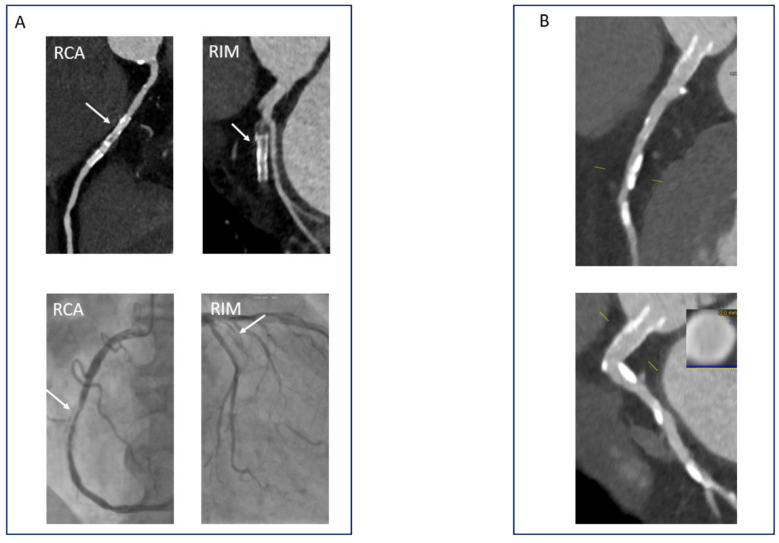
CCTA in the evaluation of coronary stents. (**A**) Note the in-stent restenosis in the RCA as well as the stent occlusion in the ramus intermedius in a patient with coronary stent placement 8 years ago (ICA images for comparison). (**B**) CCTA evaluation of a stent placed in the left main 3 years after the PCI.

In-stent restenosis (ISR) still remains a relatively rare but clinically relevant problem, even in the era of modern drug-eluting stents [161]. The evaluation of stents poses challenges for CCTA due to hard beam and blooming artefacts, motion artefacts, and inhomogeneous contrasting of the stent lumen [162]. Several studies and meta-analyses have looked at the ability of multidetector CT to identify ISR. In general, they point to a very good negative predictive value (98%) in segments that were considered assessable with CCTA [163,164]. As expected, the values for sensitivity, specificity, and positive and negative predictive values dropped when all segments were included in the analysis [165]. The main predictor for an optimal evaluation with CCTA was a stent diameter > 2.75–3 mm [166,167]. Other negative predictors for optimal evaluation of coronary stents are heavy calcification, overlapping segments, and stents with thick struts [168,169]. Thus, stents implanted in the left main or proximal segments of the coronary arteries can most appropriately be evaluated using CCTA (Figure 10A,B). Furthermore, dual source machines are preferred since they provide higher temporal resolution [170]. Finally, the use of a sharp convolution kernels may reduce the blooming artefact. 

### 6.2. Evaluation of Bypass Grafts: Looking at the Surgeon’s Work

**Figure 11:**
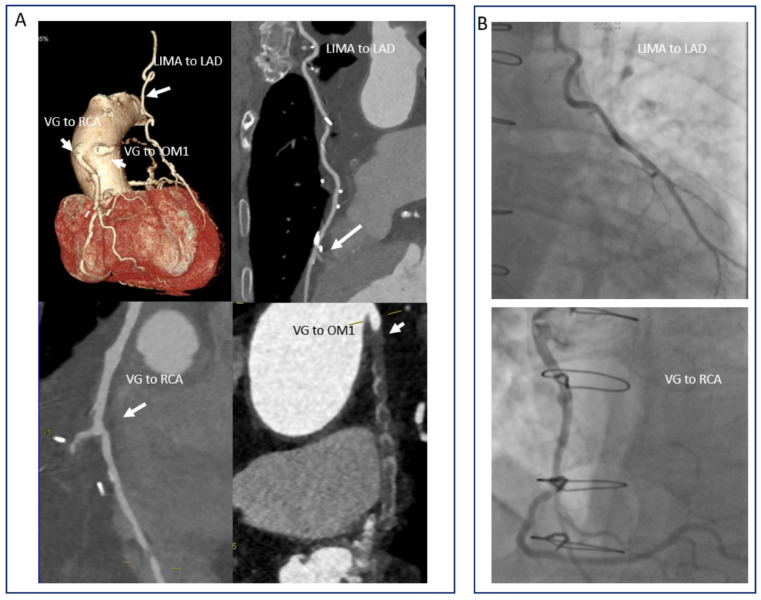
CCTA in the evaluation of coronary artery bypass patients (**A**). Note the patency of the LIMA to LAD and VG to RCA. The VG to the first obtuse marginal is occluded. Corresponding images from the coronary angiography (**B**).

Although major advances were seen in PCI technique in the last decades, coronary artery bypass graft (CABG) surgery still remains the standard of care in advanced multi-vessel CAD [171]. Depending on the type of vessel used, the patency of grafts at 10 years is 61% for venous grafts and 85% for arterial grafts [172]. CCTA is an excellent method for the evaluation of bypass grafts since they are less susceptible to motion artefacts and have larger diameter (especially venous grafts) than native vessels (Figure 11). Similar protocols to a standard CCTA can be used for the evaluation of bypass grafts [173]. However, because most CABG operations make use of the left interior mammary artery (LIMA), the acquisition window is significantly larger as it needs to include the subclavian artery. This translates into increased radiation exposure for the patient as well as longer breath-holds. Furthermore, because a larger area has to be acquired, the administration of contrast has to be optimized (i.e., larger volume or higher speed of administration) so that the distal segments of the coronary artery (runoff vessel) are adequately filled with contrast. Lastly, clips used for the LIMA can induce hard beaming or streak artefacts. All in all, the diagnostic performance of CCTA in the evaluation of bypass grafts is excellent, with studies yielding sensitivities and NPV of 99% [174,175]. The native coronary vessels are, on the other hand, more difficult to assess since these vessels are often occluded and heavily calcified. 

The value of CCTA in establishing the appropriate treatment strategy—interventional or operative—in patients with left main or three vessel coronary artery disease was tested in the SYNTAX III trial [176]. A total of 223 patients received a CCTA and invasive coronary angiography, and two separate heart teams made a recommendation related to the appropriate treatment strategy—percutaneous coronary intervention or coronary artery bypass surgery—on the basis of the value of the SYNTAX score, independent of each other. The agreement in regard to treatment strategy between CCTA and ICA was very good (kappa 0.82). Furthermore, a similar very good agreement (0.80) in regard to which coronary segments should be revascularized was found between the two heart teams. 

### 6.3. Coronary Anomalies and Muscle Bridges: Not So Rare

**Figure 12:**
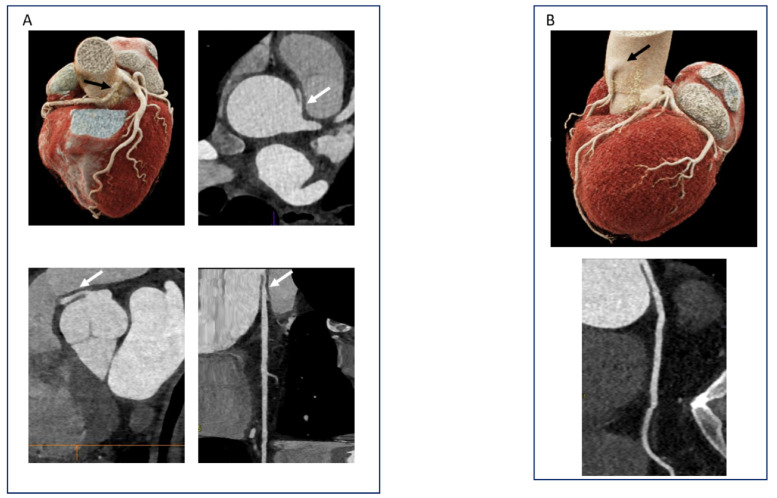
CCTA in the evaluation of coronary anomalies. (**A**). Patient with high-risk features of an RCA originating from the left coronary sinus: proximal narrowing, intramural trajectory, and course between the aorta and pulmonary artery. (**B**). Benign coronary anomaly with a high take-off of the RCA.

Coronary anomalies are a relative common finding in CCTA studies, although they affect less than 1% of the general population [177]. In most cases, they are incidental findings. However, their clinical significance ranges from benign occurrences to causes of sudden cardiac death. CCTA represents an excellent method for the visualization of coronary anomalies, as it allows for a three-dimensional reconstruction of the entire coronary tree and thus provides information related to site of origin, course of the vessel, relation to the adjacent structures (mainly the great vessels), and possible associated atherosclerotic disease, as well as, when deemed necessary, helping in planning the surgical intervention [178]. Several markers of “malignant” anomalies have been described: anomalous course of the vessel between the aorta and the pulmonary artery, intramural course, acute take-off angle, proximal narrowing, and elliptic proximal shape [179] (Figure 12A). High take-off of the coronary artery from the aorta (Figure 12B) and absence of left main with separate origins of the LAD and LCX, on the other hand, are considered to be benign anomalies [180]. Myocardial bridges are relatively often seen in CCTA as well on invasive angiograms. However, their functional significance is less certain but generally considered benign if ischemia is not induced during a functional testing [181]. Lastly, coronary fistulas represent a connection between the coronary arteries and cardiac chamber, cardiac vein, or pulmonary artery. They are incidental findings, and their functional relevance depends on the amount of flow that is “shunted” from the coronary circulation to the lower pressure system [182]. 

### 6.4. Cardiac CT in Other Clinical Scenarios: Valvular Heart Diseases and Atrial Fibrillation

The field of transcatheter interventions for valvular heart disease has seen tremendous developments in the last decades. Cardiac CT currently plays a pivotal role in planning for a transcatheter aortic valve replacement providing reliable information related to coronary anatomy, severity of the aortic valve stenosis, and accurate anatomic characterization of the aortic root [183]. Furthermore, within a single examination, valuable data related to vascular access route are also acquired. More recent studies have pointed to an important role of cardiac CT in planning for transcatheter interventions for the mitral valve [184,185]. The field of percutaneous interventions for the tricuspid valve is currently expanding. Similar to other type of valvular diseases, cardiac CT aids in planning transcatheter tricuspid valve therapies, offering valuable information related to the side of the tricuspid ring the right ventricle as well as anatomical relations between the right coronary artery and the tricuspid ring [186]. Another important field of cardiology that profits from the routine use of cardiac CT is electrophysiology and especially the peri-procedural planning for pulmonary vein isolation (PVI) in patients with atrial fibrillation [187]. PVI is currently an established therapeutic option in patients with atrial fibrillation and has been proven to improve the prognosis in patients with reduced ejection fraction due to increased heart rates [188]. Cardiac CT provides an accurate three-dimensional rendering of the left atrium and the pulmonary veins. It was shown to improve the accuracy of the ablation procedure provide information related to possible anatomic variations and reduce radiation exposure during the procedure [189,190,191]. Furthermore, using cardiac CT, cardiac thrombi can be safely excluded [192]. 

## 7. Conclusions

Coronary computer tomography angiography is an excellent method for the evaluation of patients with known or suspected coronary artery disease, with a large body of evidence to support this. It currently provides a vast array of information, both morphological and functional, and helps in establishing an appropriate treatment strategy in these patients. 

## Figures and Tables

**Table 1:** CAD-RADS classification of patients who undergo a CCTA. Adapted from Cury et al. [73].

	Maximal Coronary Artery Stenosis Severity (Per Patient)	Interpretation	Further Recommendations
CAD-RADS 0	0%	“no plaque or stenosis”	None
CAD-RADS 1	1–24%	“plaque without stenosis”	None
CAD-RADS 2	25–49%	“mild stenosis”	None
CAD-RADS 3	50–69%	“moderate stenosis”	Consider functional testing
CAD-RADS 4A	70–99%	“severe stenosis”	Consider functional testing or ICA
CAD-RADS 4B	Left main > 50% or 3-vessel disease ≥70%	“severe stenosis”	ICA is recommended
CAD-RADS 5	100%	Total coronary occlusion	ICA and/or test for viability
CAD—RADS N	Non diagnostic	CAD cannot be excluded	Consider alternative tests

## Data Availability

Not applicable.
